# Low serum klotho concentration is associated with worse cognition, psychological components of frailty, dependence, and falls in nursing home residents

**DOI:** 10.1038/s41598-021-88455-6

**Published:** 2021-04-27

**Authors:** Begoña Sanz, Haritz Arrieta, Chloe Rezola-Pardo, Ainhoa Fernández-Atutxa, Jon Garin-Balerdi, Nagore Arizaga, Ana Rodriguez-Larrad, Jon Irazusta

**Affiliations:** 1 Department of Physiology, Faculty of Medicine and Nursing, University of the Basque Country (UPV/EHU), 489040 Leioa, Bizkaia Spain; 2 Department of Nursing II, Faculty of Medicine and Nursing, University of the Basque Country (UPV/EHU), 20014 Donostia-San Sebastián, Gipuzkoa Spain; 3 Department of Didactics of Musical, Plastic and Corporal Expression, Faculty of Education, University of the Basque Country (UPV/EHU), 489040 Leioa, Bizkaia Spain; 4 Department of Nursing I, Faculty of Medicine and Nursing, University of the Basque Country (UPV/EHU), 489040 Leioa, Bizkaia Spain; 5 Caser Residencial Anaka, Fundación Caser, 20301 Irun, Gipuzkoa Spain; 6 Intensive Care Unit, Donostia University Hospital, 20014 Donostia, Spain

**Keywords:** Biomarkers, Health care, Risk factors

## Abstract

Serum alpha-klotho (s-klotho) protein has been linked with lifespan, and low concentrations of s-klotho have been associated with worse physical and cognitive outcomes. Although its significance in aging remains unclear, s-klotho has been proposed as a molecular biomarker of frailty and dependence. This study is a secondary analysis of data from a clinical trial performed in a population of 103 older individuals living in 10 nursing homes in Gipuzkoa (Spain). We aimed to elucidate associations between s-klotho (as measured by enzyme-linked immunosorbent assay) and body composition, physical fitness, and cognition, as well as frailty and dependence (determined using validated tests and scales). In addition, we investigated the association of s-klotho concentration with falls in the six months following the initial assessment. Low s-klotho levels were associated with a lower score in the psychological component of the Tilburg Frailty Indicator, a worse score in the Coding Wechsler Adult Intelligence Scale, and a greater dependence in activities of daily living. Moreover, participants with lower s-klotho concentrations suffered more falls during the 6 months after the assessment. Future translational research should aim to validate klotho’s putative role as a biomarker that could identify the risk of aging-related adverse events in clinical practice.

## Introduction

**Figure 1:**
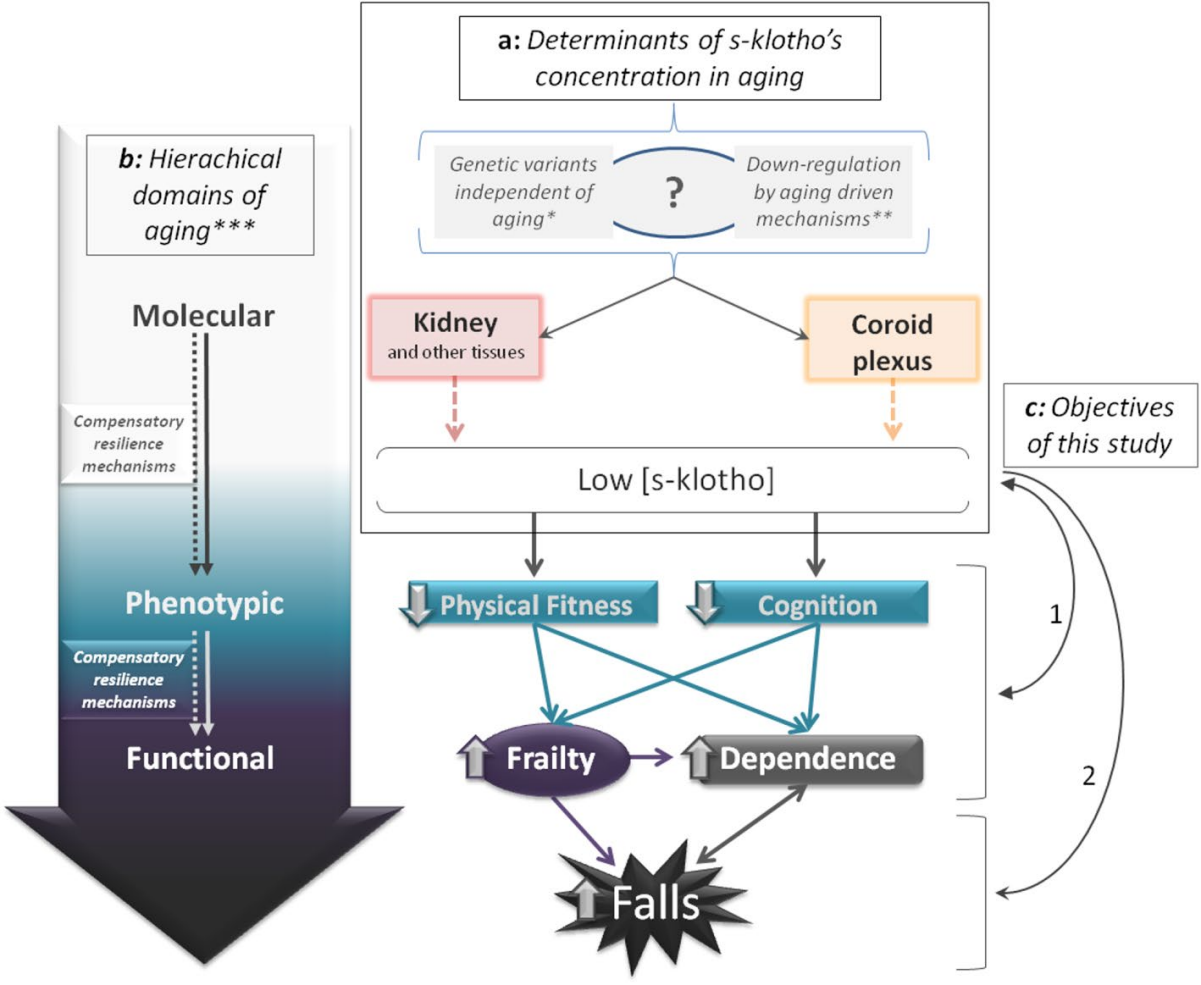
(**a**) Schematic representation of the determinants of s-klotho’s concentration in aging *^7^, **^8,9^ and (**b**) the hierarchical domains of aging ***^42^. (**c**) The objectives of this study: to ascertain the association of s-klotho (1) with some parameters linked to the phenotypic and functional domains of aging and (2) with falls in a six-month follow-up.

Alpha-klotho is a transmembrane protein that is primarily expressed in the kidney and the choroid plexus of the brain^1^ and can be cleaved and released in a soluble form (s-klotho) that acts as a hormone on tissues and cells that do not express the *klotho (KL)* gene, which encodes alpha-klotho^2^. Kuro-o et al.^3^ found that mice lacking the *KL* gene showed aging-related characteristics such as arteriosclerosis, osteoporosis, skin atrophy and emphysema and died prematurely. In the same vein, other authors have demonstrated that overexpression of *KL* can extend life span in mice^4^. In humans, s-klotho also seems to be crucial in the pathophysiology of age-related disorders^3^ and lower plasma klotho concentration has been associated with a higher risk of mortality^5^, however, its significance in aging remains to be elucidated^6^. Evidence suggests that s-klotho concentration can be determined both by genetic variations independent of aging^7^ or by downregulation via aging-driven mechanisms^8,9^ (Fig. 1a).

Although s-klotho plays a key role in energy metabolism^10^, investigation of the relationship between klotho and physical fitness is scarcer in aged populations than in other population groups^11^. However, studies about the association of s-klotho levels with physical fitness in older or middle-aged adults point in the same direction: low s-klotho has been associated with poor grip^12^, decreased lower limb strength^13,14^ and dependence^15^ in older adults and with poor physical fitness in middle-aged sedentary adults^16^.

S-klotho is also secreted in the brain, and klotho-deficient mice demonstrated brain atrophy^3^, difficulty in learning^17^, fewer hippocampal synapses, axonal transport disorders, hippocampal neurodegeneration^18^ and demyelination^19^. Accordingly, other authors found that mice overexpressing the *KL* gene showed better cognitive functioning and performance on memory tasks^19^. The involvement of klotho in calcium transport^20^ could explain its association with cognitive function^21^. In humans, lower klotho concentration has been associated with worse cognitive function^21,22^, neurodegeneration^22^, and demyelination^17^. Klotho concentration was lower in cerebrospinal fluid collected from patients with Alzheimer’s disease compared to samples from a control population^8^, and in patients with multiple sclerosis, lower klotho was associated with disability^23^. These results support the positive role of klotho in the central nervous system not only in mice, but also in humans.

Activities of daily living (ADL) are often used as predictors of health and function in older individuals^24^. Based on previous investigations from the same group showing that older adults with low serum klotho concentrations had poor skeletal muscle strength and were at a higher risk of mortality, Crasto et al.^15^ hypothesized that klotho was associated with dependence. They found for the first time that plasma klotho is independently associated with disability in ADL in community-dwelling older adults^15^.

Frailty is a geriatric syndrome that refers to an increase in vulnerability to adverse health outcomes^25,26^, including falls, and leads to a loss of autonomy and dependence^27^, diminishing individuals’ quality of life^15^. Although there are few studies on the topic, some authors have found an association between lower klotho concentration and frailty^1,28,29^, which is consistent with the role of klotho as an anti-aging hormone linked to muscle and cognition.

The risk of falling increases in older adults with low muscle strength and/or cognitive deficits^30^. In addition, people living in nursing homes tend to have higher risk^31^ and rates of falling^32^ than those living in the community^33^. Falls can result in injuries, giving rise to physical limitations, dependence^34^, psychological consequences^35^, and/or deterioration of quality of life^30^. Moreover, falls constitute a major public health problem^36^ with a high impact on the economic cost of health and social systems^37^.

Assessment of fall risk is challenging due to its complex nature^38^. Identification of molecular biomarkers related to fall events may provide an approach for identifying individuals at risk and clues to the pathophysiological mechanisms related to falls^34^. Some biomolecules closely related to klotho, such as vitamin D and fibroblast growth factor 23 (FGF-23)^39^, have been associated with the risk of falls in older populations^40,41^. However, to our knowledge, no studies have yet analyzed the ability of serum klotho concentration to predict falls.

The vast majority of published studies about klotho and aging are focused on single spheres such as physical fitness, cognition, dependence, or frailty. To the best of the authors’ knowledge, to date, no study has investigated the role of klotho on aging from an integrative perspective. Thus, the present study aims to elucidate the association of s-klotho with parameters related to several hierarchical domains of aging^42^ (Fig. 1b) in a population of older people living in nursing homes. In particular, we aimed to ascertain the association of s-klotho (1) with the phenotypic (physical fitness and cognition) and functional (frailty and dependence in ADL) domains of aging and (2) with falls in a six-month follow-up (Fig. 1c).

## Materials and methods

### Study design and participants

This is a secondary analysis of the baseline data from a randomized controlled physical exercise intervention that included a 6-month follow-up for falls. The study was conducted at ten nursing homes in Gipuzkoa, Spain (ACTRN12616001044415), between October 2016 and July 2017^43^. The study adheres to the STROBE guidelines. This study was approved by the Ethics Committee for Research in Humans (CEISH: M10/2016/105) and by the Ethics Committee for Research with Biological Agents of the University of the Basque Country (CEIAB: M30/2016/106). Written informed consent was provided by each participant. The results of this exercise intervention were published previously^44–46^.

The primary study included 112 men and women living in nursing homes who met the following criteria: ≥ 70 years old; scored ≥ 50 on the Barthel Index for Activities of Daily Living (0–100)^47^; scored ≥ 20 on the MEC-35 test (0–35), an adapted and validated version of the Mini Mental State Examination (MMSE) in Spanish^48^; and were capable of standing up and walking independently for at least ten meters. In this secondary analysis, 103 available serum samples from the 112 total participants were analyzed.

### Serum klotho concentration

Baseline blood samples were collected in the morning following an overnight fast. Following collection, the tubes were centrifuged at 5000×*g* for 10 min. The serum obtained from each participant was stored in aliquots at − 80 °C for further analysis. A commercial enzyme-linked immunosorbent assay (ELISA) was performed to measure klotho serum concentration according to the manufacturer’s protocol (Human soluble α-Klotho Assay Kit JP27998, Immuno-Biological Laboratories Co., Ltd., Gunma, Japan). The quantification was performed spectrophotometrically using a FLUOstar OPTIMA Microplate Reader (ThermoFisher Scientific, Waltham, MA, USA) and Optima Control software version 2.20 (http://www.tmd.ac.jp/cmn/gene/center/0413F0011A%20%20Software%20Manual%20OPTIMA%20Part%20II.pdf) (BMG LABTECH GmbH, Offenburg, Germany). Serum samples were measured in duplicate and averaged. Coefficient of variability (%CV) was calculated using the following formula: %CV = standard deviation/mean. Limits for %CV were set at 5% and 10% for intra-assay and inter-assay measurements. respectively. All calculated %CV values were within these limits.

### Other analyzed variables

At the baseline of the intervention, sociodemographic data (sex, age and education level) and Barthel Index scores^47^ were recorded from the database of the nursing homes. The Barthel Index reflects dependence in activities of daily living (ADL), and its score ranges from 0 to 100, with lower scores indicating a higher degree of dependence.

Regarding anthropometry, waist and hip circumference were measured with a non-elastic anthropometric tape and measured to the nearest 0.1 cm. Height was measured with a Holtain stadiometer to the nearest 0.1 cm, and body mass was measured with an Omron digital scale to the nearest 0.1 kg. Body mass index (BMI) was calculated based on height and mass, and the waist-to-hip ratio was based on waist and hip circumferences. Percentages of muscle and fat were measured using a portable bioelectrical impedance analyzer (Bodystat BIA Quadscan 4000, BOSYSTAT LTD; Isle of Man, UK). This method was supported by the European Working Group on Sarcopenia in Older People (EWGSOP) as a valid and cost-effective alternative to dual X-ray absorptiometry (DXA)^49^.

Physical fitness was assessed through the handgrip strength test using a Jamar dynamometer^50^, and aerobic capacity was evaluated with the 6-Min Walk test of the Senior Fitness Test^51^. Dynamic balance was assessed with the Timed Up and Go Test^52^, and static balance with the Berg Balance Scale^53^. Lower limb function (static balance, walking velocity, and lower limb strength) was evaluated with the Short Physical Performance Battery (SPPB)^54^.

Global cognition was assessed using the Montreal Cognitive Assessment (MoCA)^55^, which included visuospatial/executive function, naming, attention, language, abstraction, delayed recall, and orientation. Trail Making Test A^56^ was administered to evaluate executive function, and the Coding test was used to measure processing speed on the Wechsler Adult Intelligence Scale, Fourth Edition (WAIS IV)^57^. Higher scores on these scales indicate better cognitive functioning, with the exception of Trail Making Test A, in which higher scores indicate worse performance. The MoCA, Trail Making Test A and Coding cognitive tests require participants to see and draw. Therefore, participants with visual impairments or difficulties holding a pen were unable to perform (some of) these tests. Regarding Trail Making Test A, some of the participants were unable to finish the test; thus, no result was recorded. For these reasons, the number of participants in cognitive tests varies.

Frailty status was assessed with the Fried Frailty Index (FFI)^25^, the Tilburg Frailty Indicator (TFI)^58^, and the Clinical Frailty Scale (CFS)^59^. Frailty, according to the Fried’s frailty phenotype, was identified by the presence of three or more of the following signs/symptoms: unintended weight loss, exhaustion, weakness, slow gait speed, and low physical activit^25^. The TFI contains 15 questions on physical, psychological, and social domains of frailty. Participants with a score of 5 or more were considered frail according to the TFI^58^. For the CFS, frailty status is based on clinical judgment. Possible scores range from 1 to 9, and participants with a score of 6 points or higher were considered frail.

All of the tests were performed by accredited and experienced professionals in each area.

The number of falls suffered by each participant within six months after baseline was recorded from the database of the nursing homes.

### Statistical analyses

Statistical analysis was performed using SPSS version 24 (https://www.ibm.com/es-es/analytics/spss-statistics-software) (IBM, Chicago, IL, USA). The Kolmogorov–Smirnov test was used to test the normality of the data. When data were not normally distributed (s-klotho concentration, hand grip, Timed Up and Go test, all cognition tests, Barthel scale, frailty indicators), they were square root transformed before being analyzed using parametric tests. Qualitative variables are presented as n (%) and quantitative variables as mean ± SD. Due to the exploratory nature of this study, and in order to avoid type II errors, Bonferroni correction was not applied to the statistical analysis^60^.

Pearson correlations were carried out to examine associations between s-klotho concentration and quantitative variables (age, anthropometry, physical fitness, cognition, Barthel Index and frailty scores). Due to the uneven distribution of the number of falls, its association with klotho was calculated using a univariate Poisson regression. Differences in s-klotho levels between groups based on qualitative variables (sex, education level, frailty status, assignation to the control or the intervention group for the exercise program and fallers vs*.* non fallers) were assessed using Student’s t-test.

Results were considered statistically significant at p < 0.05.

### Ethics approval and consent to participate

This study was approved by the Ethics Committee for Research in Humans (CEISH: M10/2016/105) and by the Ethics Committee for Research with Biological Agents of the University of the Basque Country (CEIAB: M30/2016/106).

## Results

### Descriptive characteristics of the sample

Baseline descriptive data are shown in Table 1. The mean concentration of s-klotho was 0.97 (± 0.18) ng/ml. Of the 103 participants, 73 (70.9%) were women and 30 (29.1%) were men, with an average age of 84.7 (± 6.96) years. Regarding frailty, for each tool employed (FFI, TFI, and CFS), the mean score, as well as the frailty status (no/yes), are shown. The number of falls during the 6-month follow-up is also shown, as the number and percentage of participants who fell or not.

**Table 1:** Baseline characteristics of the studied population.

Variables	N	N	(%)
		Mean	± SD
**[s-klotho] (ng/ml)**	103	0.97	± 0.18
**Sociodemographic data**			
Sex	103		
Women		73	(70.9)
Men		30	(29.1)
Age (years)	103	84.7	± 7.0
Years of education	103		
≤ 12		95	(92.2)
> 12		8	(7.8)
**Anthropometry**			
Body mass index (kg/m^2^)	100	28.5	± 5.2
Fat (%)	100	46.7	± 8.8
Muscle (%)	100	53.3	± 8.8
Waist-to-hip ratio	100	0.98	± .08
**Physical fitness**			
Hand grip (kg)	101	20.4	± 7.9
6 min walk test (m)	101	224.7	± 96.9
Gait speed 4 m (m/s)	98	0.64	± 0.24
Timed Up and Go Test (m/s)	100	0.32	± 0.14
Berg Balance Scale (score 0–56)	98	44.7	± 8.4
Short Physical Performance Battery (score 0–12)	98	5.8	± 3.0
**Cognition**			
Montreal Cognitive Assessment (score 0–30)	83	14.0	± 1.7
Trail Making Test A (s)	57	128	± 54.4
Coding Wechsler Adult Intelligence Scale (WAIS IV; score 0–30)	77	11.9	± 8.9
**Activities of daily living**			
Barthel Index (score 0–100)	103	81	± 13
**Frailty**			
Fried Frailty Index (score 0–5)	94	2.9	± 1.1
No (< 3)		35	(37.2)
Yes (≥ 3)		59	(62.8)
Tilburg Frailty Indicator (score 0–15)	93	5.8	± 3.0
No (< 5)		35	(37.6)
Yes (≥ 5)		58	(62.4)
Physical components (score 0–8)	93	3.3	± 2.2
Psychological components (score 0–4)	93	1.7	± 0.98
Social components (score 0–3)	93	0.78	± 0.47
Clinical Frailty Scale (score 1–9)	101	5.7	± 0.79
No (≤ 6)		40	(39.60)
Yes (> 6)		61	(60.40)
**Falls (0–6 months)**	80	1.13	± 2.19
No		46	(57.50)
Yes		34	(42.50)

*SD* standard deviation.

### Association of s-klotho with quantitative variables

Table 2 shows baseline Pearson’s (Table 2A) and Poisson’s (Table 2B) correlations of s-klotho with the quantitative variables analyzed. No association was observed between s-klotho concentration and age, anthropometry, or physical fitness. Regarding cognition, participants with lower s-klotho levels showed a trend toward worse scores in cognition-related tests, with statistical significance in the coding test (WAIS IV), which reflects processing speed (r = 0.256, p = 0.012). Regarding the Barthel Index, lower s-klotho was associated with higher dependence in ADL (r = 0.253, p = 0.010). When analyzing frailty scores, the only association found was with the psychological components of the TFI, with lower klotho concentration being associated with higher scores in psychological components of frailty (r =− 0.231, p = 0.026). S-klotho was also negatively associated with the number of falls (B = − 3.311, p < 0.001).

**Table 2:** Correlations between s-klotho concentration and quantitative variables. A: Pearson correlations for sociodemographic data, anthropometry, physical fitness, cognition, activities of daily living and frailty scores. **B:** Poisson correlation for number of falls.

A					
[s-klotho] (ng/ml)	N	Pearson coefficient	p-value		
**Sociodemographic data**					
Age (years)	103	− 0.009	0.928		
**Anthropometry**					
Body mass index (kg/m^2^)	100	− 0.125	0.215		
Fat (%)	100	− 0.024	0.816		
Muscle (%)	100	0.027	0.789		
Waist-to-hip ratio	100	− 0.010	0.919		
**Physical fitness**					
Hand grip (kg)	103	0.025	0.805		
6 min walk test (m)	101	− 0.017	0.867		
Gait speed 4 m (m/s)	98	− 0.009	0.927		
Timed Up and Go Test (m/s)	100	− 0.039	0.703		
Berg Balance Scale (score 0–56)	98	0.040	0.693		
Short Physical Fitness Battery (score 0–12)	98	0.025	0.806		
**Cognition**					
Montreal Cognitive Assessment (0–30 points)	83	0.175	0.115		
Trail Making Test A (s)	57	− 0.172	0.200		
Coding Wechsler Adult Intelligence Scale (WAIS IV; 0–30 points)	77	0.286	0.012		
**Activities of daily living**					
Barthel Index	103	0.253	0.010		
**Frailty**					
Fried Frailty Index (score 0–5)	94	− 0.045	0.667		
Tilburg Frailty Indicator (score 0–11)	93	− 0.155	0.139		
Physical components (score 0–8)	93	− 0.103	0.324		
Psychological components (score 0–4)	93	− 0.231	0.026		
Social components (score 0–3)	93	0.005	0.960		
Clinical Frailty Scale (score 1–9)	101	− 0.136	0.175		

B					
Number of falls	N	Poisson coefficient	95% (CI)	Wald Chi-square	p-value
[s-klotho] (ng/ml)	80	− 3.311	(− 4.785/− 1.836)	19,369	< 0.001

*CI* Confidence interval.

### Differences in s-klotho within groups of qualitative variables

Table 3 shows the mean (± standard deviation) s-klotho concentration according to sex, education level, frailty status, assignation to the control or the intervention group for the exercise program and falling status during the 6-month follow-up. S-klotho levels were lower in participants who fell compared to those who did not fall (p = 0.037). No other statistically significant differences were found for qualitative variables.

**Table 3:** Student’s t-test comparing s-klotho concentrations in groups based on sex, years of education, frailty status, participation in the exercise program and falls.

Variables	N	[s-Klotho] (ng/ml)		t-value	Cohen’s d	p-value
		Mean	± SD			
**Sociodemographic data**						
Sex						
Women	30	0.97	± 0.18	− 0.04	− 0.01	.968
Men	73	0.97	± 0.19			
Years of education						
≤ 12	95	0.96	± 0.18	− 0.79	− 0.29	.428
> 12	8	1.01	± 0.17			
**Frailty**						
Fried Frailty Index (score 0–5)						
No (< 3)	35	0.96	± 0.18	− 3.29	− 0.07	.743
Yes (≥ 3)	59	0.98	± 0.19			
Tilburg Frailty Indicator (score 0–11)						
No (< 5)	35	0.99	± 0.20	1.13	− 0.24	.263
Yes (≥ 5)	58	0.95	± 0.16			
Clinical Frailty Scale (score 1–9)						
No (< 6)	40	1.00	± 0.21	1.26	0.27	.213
Yes (≥ 6)	61	0.95	± 0.15			
**Intervention group**						
No	52	0.99	± 0.19	1.25	0.25	.215
Yes	51	0.94	± 0.17			
**Falls (0–6 months)**						
No	46	0.99	± 0.16	2.12	0.07	.037
Yes	34	0.92	± 0.15			

*SD* standard deviation.

## Discussion

In this study, we have analyzed serum klotho concentrations in a population of 103 older adults living in nursing homes. Lower s-klotho concentration was associated with lower processing speed (higher score in the coding test [WAIS IV]) and more dependence in ADL (lower score in the Barthel Index). In addition, participants with lower s-klotho levels showed a worse score in the psychological components of the TFI. However, no association was found with any other scores on frailty assessment tools, nor were any differences found between the frail and non-frail participants in any of the tools used. Participants with lower s-klotho levels suffered more falls in the six-month follow-up.

Due to the described role of klotho in preventing muscle loss^1^, it could be predicted to be positively associated with parameters related to muscle and physical fitness. However, in our work, neither body composition nor scores in physical performance tests were associated with s-klotho. Other authors have described an association between lower s-klotho and less muscle mass^16^. The disagreement between their results and those of our study could be due to the population studied (middle-aged sedentary adults vs*.* older people living in nursing homes) or in the method employed to determine body composition (DXA vs. bioelectrical impedance analyzer).

Regarding physical performance, lower s-klotho has been associated with less strength^12,13^ and worse results in tests of physical fitness^14,15,61^. This discordance between these results and ours could be due to the different populations under study, as these previous studies were conducted in general samples consisting of younger and fitter community-dwelling middle-aged^14,16^ or older adults^12–15^. To our knowledge, no study on the analysis of s-klotho levels in populations living in nursing homes has yet been published.

Although the association of klotho with cognition is not yet well established in humans^15^, lower s-klotho concentration has been associated with worse global cognitive function^15,21^ and higher risk of cognitive decline^21^. In our study, participants with lower serum s-klotho concentrations obtained worse results in cognition-related tests, although this association was only significant for the coding test. The coding test measures processing speed^57^, whereas the Trail Making Test A reflects executive function^56^. Similar results that linked global cognitive function but not executive function with s-klotho have been described by Shardell et al.^21^ in community-dwelling older adults. Better cognition associated with enhanced expression of klotho has been demonstrated in animal models^3,7^ as well as in humans^8,15,17,21,22^; this is likely linked to the action of brain-secreted klotho on the structure of the synapses^18^. Although the mechanisms underlying klotho’s regulation in the brain are still unknown, klotho has been proposed as a potential target for therapies aimed at cognitive enhancement^62^.

Based on klotho’s functions and some preclinical and clinical evidence, Angulo et al.^9^ have proposed that its upregulation would prevent phenotypic characteristics of frailty in the elderly. Loss of function of serum s-klotho has also been described as a quantifiable biomarker on the continuum between frailty and resilience^28^ and for frailty diagnosis and stratification^28^. However, we did not observe differences in serum s-klotho concentrations between frail and non-frail older people living in nursing homes.

Despite the lack of association between klotho and frailty in our study, participants with lower s-klotho levels exhibited lower scores in the psychological components of the TFI. Psychological frailty encompasses cognitive, mood, and motivational components^63^, and some authors have found its association with mental health-related quality of life^64^. In addition, De Roeck et al.^65^ have recently proposed that cognitive frailty can occur independent of the other frailty domains, including physical frailty, and can therefore be seen as a distinct concept. These concepts and findings are in line with our result, in which s-klotho was associated with cognition and psychological components of the TFI, but not with the physical or clinical tools for identifying frailty.

The lack of significant associations of s-klotho with muscle-related variables and frailty could be explained by the suspicion that the biology and functions of klotho may be more complex than previously hypothesized^6,28,66^. Thus, compensatory/resilient mechanisms could mask the consequences of changes in molecular levels on the phenotypic and functional responses^42^. Moreover, most of the published studies on klotho are related to its serum concentration or its genetic expression. However, klotho is a multifunctional protein with enzymatic activity as a glucuronidase (EC: 3.2.1.31) hydrolyzing extracellular sugars and has great importance in signaling pathways^67^. As it is able to perform multiple functions (hormone, enzyme, and receptor)^66^ that differ mechanistically^68^, klotho could be considered a moonlighting enzyme. This could have consequences in the comprehension of its regulatory mechanisms and supposed functions and implications for the functionality of the organism.

Our results also show that individuals with lower s-klotho concentrations were more dependent in their performance of ADL. Similar results have been published by Crasto et al.^15^ in a population of community-dwelling older men and women. The loss of independence is one of the ultimate consequences of age-associated decline, but the changes that occur with aging, from the molecular to the functional level, are interconnected in a hierarchical arrangement. Eventually, these changes are reflected at the physical, cognitive, and emotional levels, as well as in social functions and ADL, as a result of the interaction between entropic and compensatory mechanisms^42^. However, literature about age-associated dependence and molecular biomarkers is scarce, and further insight is needed into possible biological mechanisms that may underlie risk of disability^15^.

We found an independent association of participants with a lower concentration of s-klotho at baseline and falls throughout the six-month follow-up. This association was apparent both when analyzing the number of falls and when analyzing whether participants fell or not. Falls have a significant impact on health outcomes in the elderly and are associated with various factors, including impairments in cognition and dependence in ADL^38^. Due to the consequences of falling, identifying fall prediction tools might lead to novel translational approaches from basic research to clinical practice.

Few studies have focused on the relationship between molecular biomarkers and falls^34^, and to our knowledge, no study has yet analyzed the relationship between klotho and the risk of falling. However, there is evidence for associations between falling risk and two molecules closely related to klotho, vitamin D and FGF-23^39^, that could support our results. Vitamin D promotes secretion of circulating klotho hormone^39^, and the metabolism of these molecules is tightly associated^39,67,69^. Ensuring adequate vitamin D levels has been proposed as a strategy to reduce falls, although the results of studies investigating whether vitamin D supplementation can reduce the number of falls are inconsistent^36,40^. Indeed, it has been shown that community-dwelling older women with lower serum vitamin D concentrations suffered more falls despite the absence of an association between physical performance and vitamin D concentration^70^. These results are in line with the lack of an association between s-klotho levels and physical fitness -related parameters in our study, despite the former’s association with falls. Thus, as Uusi-Rasi et al. proposed for vitamin D^70^, s-klotho could modulate individual fall risk, albeit partially. In contrast, FGF-23 is a bone-derived hormone that inhibits α-hydroxylase, reducing serum 1, 25-dihidroxyvitamin D^71^, and α-klotho is a co-receptor for FGF-23 in the kidney^39^. Although FGF-23 has anti-aging effects, it also has some "off-target" actions that could actually be pro-aging^72^. It has been proposed that these off-target FGF-23 pathologies are opposed by secreted klotho^39^. Higher serum FGF-23 levels were associated with an increased risk of falls in patients with chronic kidney disease^41^. The opposite associations of klotho and FGF-23 with falls are in line with the opposing actions of klotho and FGF-23. Overall, these associations between biomolecules and falls support a role for the vitamin D/FGF-23/klotho system, which has been described as crucial for healthy aging, in the assessment of the risk of falls^39^.

This study has some limitations, as the small sample size, the lack of information about the medications the participants were taking, and any cognitive decline during the 6-month follow-up could act as confounders in the analysis. Due to the exploratory nature of this study and in order to increase statistical power, Bonferroni correction was not applied to the statistical analysis. However, this omission may have increased the chance of type I error. Moreover, although the bioelectrical impedance analyzer is accepted by the EWGSOP for body composition measurement and is an economic and portable technology, we recognize that more accurate results could have been obtained if DXA had been used for body composition calculation. In addition, the analysis of other molecular biomarkers related to klotho metabolism, such as FGF-23 or vitamin D, could have shown a possible interaction as molecules implicated in the regulation of the physiopathology of falling risk.

Nonetheless, this study presents a novel analysis of serum klotho and its association with future falls, presenting a line of research that could be further pursued. The introduction of a low-cost, easily standardized diagnostic tool to estimate the probability of developing falls in the near future would be very useful for routine clinical practice. Promotion of research models from the molecular mechanisms to the functional domains of aging will provide valuable information on both the molecular pathway regulation level and the functional level, which is linked to successful aging. Last, but not least, the studied population should be considered a strength of this work, since people living in nursing homes have specific characteristics and high vulnerability and comprise a poorly studied population. In addition, this study provides knowledge from an integrative perspective on aging-related outcomes, from the molecular to the phenotypic and functional domains of aging.

In conclusion, in a population of nursing home residents, lower s-klotho concentration was associated with worse cognition, scores indicating frailty in the psychological domain of the TFI, higher dependence in ADL, and future falls. These findings provide new and valuable information about the supposed role of klotho as a molecular biomarker linked to aging-related adverse events. Further investigation should be conducted to validate the putative ability of klotho to predict falling. This future research should include panels of associated molecular biomarkers that could be easily translated into clinical practice.

## References

[1] Shardell M (2019). Plasma klotho and frailty in older adults: Findings from the InCHIANTI study. J. Gerontol. A Biol. Sci. Med. Sci..

[2] Xu Y, Sun Z (2015). Molecular basis of Klotho: From gene to function in aging. Endocr. Rev..

[3] Kuro-o M (1997). Mutation of the mouse klotho gene leads to a syndrome resembling ageing. Nature.

[4] Kurosu H (2005). Suppression of aging in mice by the hormone Klotho. Science.

[5] Semba RD (2011). Plasma klotho and mortality risk in older community-dwelling adults. J. Gerontol. A Biol. Sci. Med. Sci..

[6] Koyama D (2015). Soluble αKlotho as a candidate for the biomarker of aging. Biochem. Biophys. Res. Commun..

[7] Dubal DB (2014). Life extension factor klotho enhances cognition. Cell Rep..

[8] Semba RD (2014). Klotho in the cerebrospinal fluid of adults with and without Alzheimer's disease. Neurosci. Lett..

[9] Angulo J, El Assar M, Rodríguez-Mañas L (2016). Frailty and sarcopenia as the basis for the phenotypic manifestation of chronic diseases in older adults. Mol. Aspects. Med..

[10] Razzaque MS (2012). The role of Klotho in energy metabolism. Nat. Rev. Endocrinol..

[11] Amaro-Gahete FJ (2018). Role of exercise on s-klotho protein regulation: a systematic review. Curr. Aging. Sci..

[12] Semba RD (2012). Relationship of low plasma klotho with poor grip strength in older community-dwelling adults: The InCHIANTI study. Eur. J. Appl. Physiol..

[13] Semba RD (2016). Low plasma klotho concentrations and decline of knee strength in older adults. J. Gerontol. Ser. A Biol. Sci. Med. Sci..

[14] Shardell M (2015). Serum 25-hydroxyvitamin D, plasma klotho, and lower-extremity physical performance among older adults: findings from the InCHIANTI study. J. Gerontol. A Biol. Sci. Med. Sci..

[15] Crasto CL (2012). Relationship of low-circulating "anti-aging" klotho hormone with disability in activities of daily living among older community-dwelling adults. Rejuvenation Res..

[16] Amaro-Gahete FJ (2019). Association of physical activity and fitness with s-klotho plasma levels in middle-aged sedentary adults: the FIT-AGEING study. Maturitas.

[17] Torbus-Paluszczak M, Bartman W, Adamczyk-Sowa M (2018). Klotho protein in neurodegenerative disorders. Neurol. Sci..

[18] Shiozaki M (2008). Morphological and biochemical signs of age-related neurodegenerative changes in klotho mutant mice. Neuroscience.

[19] Chen CD (2013). The antiaging protein Klotho enhances oligodendrocyte maturation and myelination of the CNS. J. Neurosci..

[20] Imura A (2007). alpha-Klotho as a regulator of calcium homeostasis. Science.

[21] Shardell M (2016). Plasma klotho and cognitive decline in older adults: Findings from the InCHIANTI study. J. Gerontol. A Biol. Sci. Med. Sci..

[22] Cararo-Lopes MM, Mazucanti CHY, Scavone C, Kawamoto EM, Berwick DC (2017). The relevance of α-KLOTHO to the central nervous system: Some key questions. Ageing Res. Rev..

[23] Emami Aleagha MS (2015). Decreased concentration of Klotho in the cerebrospinal fluid of patients with relapsing–remitting multiple sclerosis. J. Neuroimmunol..

[24] Hopman-Rock M, van Hirtum H, de Vreede P, Freiberger E (2019). Activities of daily living in older community-dwelling persons: A systematic review of psychometric properties of instruments. Aging Clin. Exp. Res..

[25] Fried LP (2001). Frailty in older adults: Evidence for a phenotype. J. Gerontol. A Biol. Sci. Med. Sci..

[26] Topinková E (2008). Aging, disability and frailty. Ann. Nutr. Metab..

[27] Hoogendijk EO (2019). Frailty: Implications for clinical practice and public health. Lancet.

[28] Abdelmalik PA (2018). Anti-aging factor, serum alpha-klotho, as a marker of acute physiological stress, and a predictor of ICU mortality, in patients with septic shock. J. Crit. Care..

[29] Lichtenauer M, Altwein AK, Kopp K, Salmhofer H (2020). Uncoupling fate: Klotho—goddess of fate and regulator of life and ageing. Australas. J. Ageing..

[30] Laurence BD, Michel L (2017). The fall in older adults: Physical and cognitive problems. Curr. Aging Sci..

[31] Vlaeyen E (2015). Characteristics and effectiveness of fall prevention programs in nursing homes: A systematic review and meta-analysis of randomized controlled trials. J. Am. Geriatr. Soc..

[32] Huang C (2016). High serum adiponectin levels predict incident falls among middle-aged and older adults: A prospective cohort study. Age Ageing..

[33] Rapp K, Becker C, Cameron ID, König HH, Büchele G (2012). Epidemiology of falls in residential aged care: Analysis of more than 70,000 falls from residents of Bavarian nursing homes. J. Am. Med. Dir. Assoc..

[34] Britton GB (2019). Inflammatory biomarkers, depressive symptoms and falls among the elderly in Panama. Curr. Aging Sci..

[35] Marschollek M (2011). Sensor-based fall risk assessment—an expert 'to go'. Methods Inf. Med..

[36] Aloia JF (2019). Vitamin D and falls in older African American women: The PODA randomized clinical lrial. J. Am. Geriatr. Soc..

[37] Balzer K, Bremer M, Schramm S, Lühmann D, Raspe H (2012). Falls prevention for the elderly. GMS Health Technol. Assess..

[38] Rubenstein LZ (2006). Falls in older people: Epidemiology, risk factors and strategies for prevention. Age Ageing..

[39] Haussler MR (2012). The role of vitamin D in the FGF23, klotho, and phosphate bone-kidney endocrine axis. Rev. Endocr. Metab. Disord..

[40] Wu H, Pang Q (2017). Einfluss der Vitamin-D- und Kalziumsupplementierung auf Stürze bei älteren Erwachsenen: Eine systematische Übersicht und Metaanalyse. (The effect of vitamin D and calcium supplementation on falls in older adults: a systematic review and meta-analysis). Orthopade..

[41] Jovanovich A (2020). FGF,23, frailty, and falls in SPRINT. J. Am. Geriatr. Soc..

[42] Ferrucci L, Levine ME, Kuo PL, Simonsick EM (2018). Time and the metrics of aging. Circ. Res..

[43] Rodriguez-Larrad A (2017). Effectiveness of a multicomponent exercise program in the attenuation of frailty in long-term nursing home residents: study protocol for a randomized clinical controlled trial. BMC Geriatr..

[44] Arrieta H (2019). Serum myostatin levels are higher in fitter, more active, and non-frail long-term nursing home residents and increase after a physical exercise intervention. Gerontology.

[45] Arrieta H (2019). Effects of multicomponent exercise on frailty in long-term nursing homes: A randomized controlled trial. J. Am. Geriatr. Soc..

[46] Arrieta H (2020). The impact of physical exercise on cognitive and affective functions and serum levels of brain-derived neurotrophic factor in nursing home residents: a randomized controlled trial. Maturitas.

[47] Wade DT, Collin C (1988). The Barthel ADL Index: A standard measure of physical disability?. Int. Disabil. Stud..

[48] Lobo A (1999). Revalidación y normalización del Mini-Examen Cognoscitivo (primera versión en castellano del Mini-Mental Status Examination) en la población general geriátrica. (Revalidation and normalization of the Mini-Cognitive Examination (first Spanish version of the Mini-Mental Status Examination) in the general geriatric population). Clin. (Barc).

[49] Cruz-Jentoft AJ (2010). Sarcopenia: European consensus on definition and diagnosis: Report of the European Working Group on Sarcopenia in Older People. Age Ageing.

[50] Fess, E. E. Grip strength in *Clinical assessment recommendations* (ed. Casanova, J.S.) 41–45 (American Society of Hand Therapists, 1992).

[51] Rikli RE, Jones CJ (2001). Senior Fitness Test.

[52] Mathias S, Nayak US, Isaacs B (1986). Balance in elderly patients: the “get-up and go” test. Arch. Phys. Med. Rehabil..

[53] Berg KO, Wood-Dauphinee SL, Williams JI, Maki B (1992). Measuring balance in the elderly: Validation of an instrument. Can. J. Publ. Health..

[54] Guralnik JM (1994). A short physical performance battery assessing lower extremity function: Association with self-reported disability and prediction of mortality and nursing home admission. J. Gerontol..

[55] Coen RF, Robertson DA, Kenny RA, King-Kallimanis BL (2016). Strengths and limitations of the MoCA for assessing cognitive functioning findings from a large representative sample of Irish older adults. J. Geriatr. Psychiatry Neurol..

[56] Reitan RM, Wolfson D (1985). The Halstead-Reitan Neuropsychological Test Battery. Theory and Clinical Interpretation.

[57] Wechsler D (2010). WAIS-IV UK Administration and Scoring Manual.

[58] Gobbens RJ, van Assen MA, Luijkx KG, Wijnen-Sponselee MT, Schols JM (2010). The tilburg frailty indicator: Psychometric properties. J. Am. Med. Dir. Assoc..

[59] Rockwood K (2005). A global clinical measure of fitness and frailty in elderly people. CMAJ.

[60] Armstrong RA (2014). When to use the Bonferroni correction. Ophthalm. Physiol. Opt..

[61] Valenzuela PL (2019). Physical performance, plasma s-klotho, and all-cause mortality in elderly dialysis patients: A prospective cohort study. Exp. Gerontol..

[62] Welberg L (2014). Cognition: Klotho spins cognitive fate. Nat. Rev. Neurosci..

[63] Fitten LJ (2015). Psychological frailty in the aging patient. Nestle Nutr. Inst. Workshop Ser..

[64] Zhang X (2019). Association between physical, psychological and social frailty and health-related quality of life among older people. Eur. J. Public Health..

[65] De Roeck EE (2020). Exploring cognitive frailty: Prevalence and associations with other frailty domains in older people with different degrees of cognitive impairment. Gerontology.

[66] Erben RG (2016). Update on FGF23 and klotho signaling. Mol. Cell Endocrinol..

[67] Chang Q (2005). The beta-glucuronidase klotho hydrolyzes and activates the TRPV5 channel. Science.

[68] Huberts DHEW, Ida J, van der Klei IJ (2010). Moonlighting proteins: An intriguing mode of multitasking. Biochim. Biophys. Acta..

[69] Ellidag HY (2016). The three sisters of fate in multiple sclerosis: Klotho (clotho), fibroblast growth factor-23 (lachesis), and vitamin D (atropos). Ann. Neurosci..

[70] Uusi-Rasi K (2019). Serum 25-hydroxyvitamin D levels and incident falls in older women. Osteoporos. Int..

[71] Shimada T (2004). Targeted ablation of FGF23 demonstrates an essential physiological role of FGF23 in phosphate and vitamin D metabolism. J. Clin. Invest..

[72] Rodelo-Haad C (2019). FGF23, biomarker or target?. Toxins..

